# Plasma visfatin levels and mRNA expression of visfatin in peripheral blood mononuclear cells and peripheral blood monocyte-derived macrophages from normal weight females with polycystic ovary syndrome

**DOI:** 10.3892/etm.2014.1556

**Published:** 2014-02-18

**Authors:** JING ZHANG, LINGLING ZHOU, LIULIN TANG, LIANGZHI XU

**Affiliations:** 1 Department of Obstetrics and Gynecology, West China Second University Hospital, Sichuan University, Chengdu, Sichuan 610041, P.R. China; 2 Department of Obstetrics and Gynecology, People’s Hospital of Yicheng, Yicheng, Hubei 441400, P.R. China; 3 Department of Obstetrics and Gynecology, Affiliated Hospital of Guilin Medical University, Guilin, Guangxi 541001, P.R. China

**Keywords:** polycystic ovary syndrome, insulin resistance, visfatin, hirsutism, infertility

## Abstract

Polycystic ovary syndrome (PCOS) is a common reproductive endocrinology disease, however, an explicit etiology is not known. Insulin resistance (IR) appears to be central to the pathogenesis of PCOS and inflammation may be significant in the pathogenesis of IR in PCOS. The aims of the present study were to investigate the plasma visfatin level and the gene expression of visfatin in peripheral blood mononuclear cells (PBMCs) and peripheral blood monocyte-derived macrophages (PBMMs) from PCOS patients, in addition to investigating the association between PCOS and IR. A total of 21 PCOS patients and 21 control subjects were enrolled in the study; the homeostasis model assessment of insulin resistance (HOMA-IR) was considered to be a stratified method for establishing the subgroups. Fasting blood samples were collected and the levels of sex hormones, insulin, glucose, blood lipids and visfatin were measured. In addition, visfatin gene expression levels in PBMCs and PBMMs were assessed using quantitative polymerase chain reaction. The plasma visfatin and gene expression levels of visfatin in PBMCs and PBMMs were not observed to increase in the normal weight PCOS and normal weight IR patients. Furthermore, plasma visfatin levels did not correlate with the normal weight PCOS patients or the normal weight IR patients *per se*. Further investigation into the role of visfatin in the pathogenesis of PCOS or IR should examine macrophages in the tissues, rather than macrophages in the peripheral blood.

## Introduction

Polycystic ovary syndrome (PCOS) is a common reproductive endocrinology disease affecting 5–10% of females of reproductive age (1). Endocrine, reproductive and metabolic abnormalities are involved in PCOS with symptoms such as infertility, irregular menstrual cyclicity, hirsutism, acne, obesity, impaired glucose tolerance, type 2 diabetes mellitus and dyslipidemia.

Previous investigations have addressed the possibility that insulin resistance (IR) and hyperinsulinemia may be central to the pathogenesis of PCOS (2). Moreover, PCOS is considered to be a low-grade chronic inflammatory state, as evidenced by the elevated plasma concentrations of numerous inflammatory factors, including tumor necrosis factor-α, C-reactive protein and interleukin-6 (3–5). IR may be induced by inflammatory cytokines through the direct or indirect action on insulin signaling postreceptor molecules in PCOS (6,7). Therefore, inflammation may play a key role in the pathogenesis of IR in PCOS.

Visfatin, a proinflammatory cytokine, is located in the visceral adipose tissue and is predominantly produced by macrophages. Injection of visfatin into mice was shown to induce a reduction in the levels of blood glucose (8). Furthermore, visfatin may mimic the function of insulin and interfere with the signal transduction of insulin (9). However, the binding point of visfatin on the insulin receptors differs from that of insulin. Visfatin reacts slowly to glucose stimulation, while insulin reacts quickly. The precise function of visfatin in humans remains unclear and the plasma visfatin levels in IR-related diseases, including obesity and type 2 diabetes mellitus, are controversial. In previous studies, increased levels of plasma visfatin were observed when PCOS patients were compared with control subjects (10–18). However, additional studies indicated that there was no difference in plasma visfatin levels between PCOS patients and control subjects, specifically between normal weight PCOS patients and control subjects (19,20). Furthermore, the association between plasma visfatin and IR in PCOS is controversial, with a positive correlation being demonstrated in a number of studies (11,17,21,22), but not in others (20,23). Obesity may have been the confounding factor that influenced those results.

Previous studies have reported an increase in mRNA expression levels of visfatin from peripheral blood mononuclear cells (PBMCs) of type 2 diabetes mellitus patients (24) and in omental adipose tissue and PBMCs of PCOS patients (17,25). However, only the visfatin mRNA concentration in the omental adipose tissue, but not the mRNA concentration in PBMCs, was closely correlated with body mass index (BMI) and the homeostasis model assessment of IR (HOMA-IR) (25). Visfatin is predominantly expressed in the macrophages of adipose tissue; however, the aforementioned study examined adipose tissue and PBMCs, rather than macrophages. Visfatin gene expression levels in the macrophages of PCOS patients have not previously been investigated to the best of our knowledge. Therefore, the correlation between gene expression of visfatin and IR in PCOS patients remains unclear.

The aim of the present study was to evaluate plasma visfatin and visfatin gene expression levels in PBMCs and peripheral blood monocyte-derived macrophages (PBMMs) of PCOS patients. The association between PCOS *per se* and IR in PCOS was also investigated.

## Patients and methods

### Patient selection

Sample size was calculated based on the results of a previous study, in which the participants were stratified into four subgroups based on their insulin sensitivity and the levels of visfatin mRNA, which were observed in the omental adipose tissue (26). A minimum of eight participants were required for each subgroup (I type error=0.05, II type error=0.1). In total, 21 PCOS patients from the reproductive endocrinology clinic in West China Second University Hospital, Chengdu, China were enrolled in the experimental group. The Rotterdam criteria of PCOS were applied (27) and patients exhibiting congenital adrenal hyperplasia, Cushing’s syndrome, androgen-secreting tumors, thyroid disease and prolactinoma were excluded. In the 21 PCOS patients, 11 were diagnosed as IR and 10 patients exhibited normal insulin levels; IR was defined as a HOMA-IR score of >2.14 (28,29).

A total of 21 patients exhibiting fallopian tube infertility, identified by a hysterosalpingogram, were recruited as control subjects and cases of polycystic ovaries and hyperandrogenism were excluded. Of the 21 controls, 9 were diagnosed as IR. Regular ovulation, identified by a normal serum progesterone level and a regular menstrual cycle, was assessed in the 12 control subjects without IR.

Participants exhibiting other infectious, organic, endocrine or systemic abnormalities were excluded from the study. The study participants did not receive medication or hormones that may have affected hormone or carbohydrate metabolism for at least three months prior to participating in the study. The study was approved by the Human Ethics Committee of West China Second University Hospital (Chengdu, China) and informed consent was obtained from all the participants.

The medical history of the participants was collected via predesigned questionnaires. Body weight, height, BMI, waist circumference, hip circumference, waist to hip ratio (WHR), and systolic and diastolic blood pressure (DBP) were measured. Cases of hirsutism, acne, acanthosis nigricans and baldness were assessed by professional analysts. The collection of blood samples was performed during the early follicular phase of the menstrual cycle (day 3–7) or following a minimum of three months of amenorrhea.

### Measurement of hormone levels

Overnight fasting blood samples were collected from all the participants. The samples were immediately centrifuged for plasma separation and stored at −80°C until the assays were conducted. Estradiol, progesterone, testosterone (T), luteinizing hormone (LH), follicle stimulating hormone (FSH), cortisol, prolactin and fasting insulin (FINS) were measured via chemiluminescence. Fasting glucose (FPG) and dehydroepiandrosterone sulfate (DHEAS) were measured using the glucose oxidase method and radioimmunoassay, respectively. Total cholesterol, triglyceride, high density lipoprotein cholesterol, low density lipoprotein cholesterol, thyronine and thyroxine were measured by enzyme-linked immunosorbent assay (ELISA). All the aforementioned tests were performed by a laboratory professional in the clinical test center of West China Second University Hospital. The inter- and intra-assay coefficient of variation were <15 and <6%, respectively. Plasma visfatin was measured using an ELISA kit (USCN Life Science Inc., Wuhan, China), with a lower limit of sensitivity of 0.78 ng/ml (range, 3.12–200 ng/ml). The inter- and intra-assay coefficients of variation were <14 and <5%, respectively.

### Ficoll gradient centrifugation

Ficoll gradient centrifugation was conducted to obtain PBMCs from the whole blood. Heparinized blood was mixed with 20 ml phosphate-buffered saline (PBS), layered onto Ficoll-Hypaque (TBD Science, Tianjin, China) and centrifuged for 20 min at 2,500 rpm (TDL-40B low-speed horizontal centrifuge, ANTING Scientific Instrument Plant, Shanghai, China). The interface containing the mononuclear cells was collected and washed three times using PBS. The cells were resuspended at 1×10^6^ cells/ml in RPMI 1640 medium (1% penicillin/streptomycin and 10% new-born calf serum) and seeded into 6-well plates at 37°C in a 5% CO_2_ humidified incubator. After 12 h, the non-adherent cells were removed and a number of the remaining PBMCs were cultured in RPMI 1640 for 96 h to obtain RNA. Additional PBMCs were cultured in RPMI 1640 with 100 nmol/l phorbol-12-myristate-13-acetate (Sigma-Aldrich, St. Louis, MO, USA) for 48 h to obtain monocyte-derived macrophages and the RNA was isolated after 96 h.

### qPCR

Total RNA was isolated from the cells using TRIzol reagent (Invitrogen Life Technologies, Carlsbad, CA, USA), with 7 μl total RNA undergoing reverse transcription in a 20-μl volume oligo dT_12–18_ Primer, according to the manufacturer’s instructions for the SuperScript^®^ III First-Strand cDNA Synthesis system (Invitrogen Life Technologies). A reverse transcribed reaction (1 μl aliquot) served as the template in a 20 μl PCR, which contained 0.2 μl per primer, 9.6 μl ddH_2_O and 9 μl 2.5X RealMaster SYBR Green I mix (Tiangen Biotech, Beijing, China) for visfatin and 0.4 μl per primer, 9.2 μl ddH_2_O and 9 μl 2.5X RealMaster SYBR Green I mix for β-actin. qPCR analysis was performed in a fluorescent temperature cycler (Mastercycler^®^ ep realplex; Eppendorf, Hamburg, Germany). Initial denaturation was conducted at 95°C for 2 min and the subsequent reactions were cycled 35 times using the following parameters to enable visfatin detection: Denaturation at 95°C for 15 sec, primer annealing at 62.7°C for 15 sec and primer extension at 68°C for 20 sec. The human visfatin oligonucleotide primers were as follows: Sense, 5′-aagagactgctggcatagga-3′ and antisense, 5′-accacagatacaggcactga-3′. mRNA detection of human β-actin was conducted as follows: Denaturation at 95°C for 2 min, 40 cycles at 95°C for 15 sec, primer annealing at 60°C for 15 sec and extension at 68°C for 20 sec. The human β-actin oligonucleotide primers were as follows: Sense, 5′-tgacgtggacatccgcaaag-3′ and antisense, 5′-ctggaaggtggacagcgagg-3′. The lengths of the qPCR products for visfatin and β-actin were 228 and 205 bp, respectively. Gel electrophoresis and melting curve analysis were applied to confirm the amplification specificity of the qPCR products from each primer pair. Standard curve methods were used to obtain the concentration of the samples and the relative visfatin mRNA levels were standardized against those of β-actin.

### Statistical analysis

The Shapiro-Wilk test was used to identify whether the variables were normally distributed and Napierian logarithm transformation was performed for specific variables, including plasma visfatin. Numerical variables are presented as the mean ± SD and differences between the groups were analyzed by one-way analysis of variance, followed by Scheffé’s method or the Games-Howell test for multiple comparisons. The paired t-test was used to analyze the difference between PBMCs and the PBMMs and Pearson or Spearman correlations were used to determine the correlation between the variables. The computations were performed using SPSS 16.0 (SPSS, Inc., Chicago, IL, USA) P<0.05 was considered to indicate a statistically significant difference.

## Results

### Participant characteristics

The clinical, hormonal and metabolic parameters for the PCOS patients and the control subjects are listed in Table I. PCOS patients were younger than the control subjects (P=0.004), however, exhibited higher DBP (P=0.03). The control non-IR participants demonstrated lower LH and LH/FSH values when compared with the other three subgroups (P≤0.003). PCOS-IR patients exhibited higher T levels than those participants in the control IR (P=0.006) and control non-IR (P=0.011) groups. In addition, PCOS-IR patients demonstrated lower FSH levels (P=0.037), but higher DHEAS (P=0.04) and FINS (P=0.016) concentrations than participants in the control non-IR group. As predicted, the IR patients exhibited higher HOMA-IR and quantities of triglyceride than the non-IR participants (P<0.001). The levels of the other indexes between the subgroups were comparable (P>0.05).

### Comparison of plasma visfatin levels

**Figure 1:**
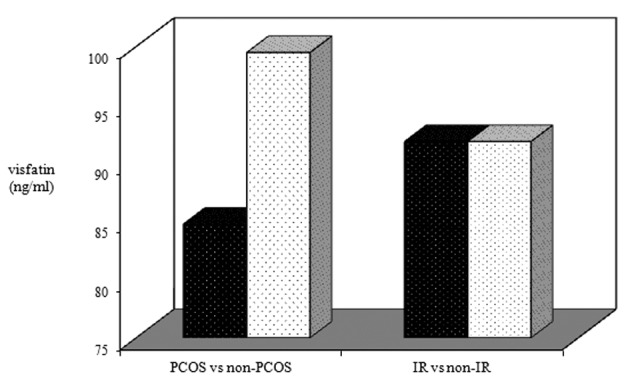
Statistical comparison of plasma visfatin levels using the Napierian logarithm transformation; no statistically significant differences were identified between the groups. PCOS, polycystic ovary syndrome; IR, insulin resistance.

**Figure 2:**
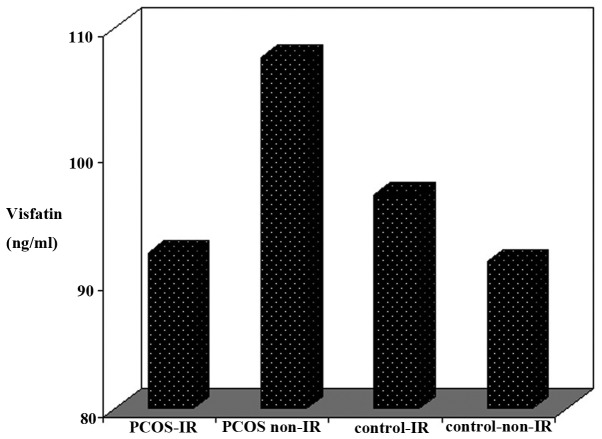
Statistical comparison of plasma visfatin levels using the Napierian logarithm transformation; no statistically significant differences were identified between the groups. PCOS, polycystic ovary syndrome; IR, insulin resistance.

No statistically significant difference was identified in the plasma visfatin levels of the participants with and without PCOS (84.77±1.35 vs. 99.48±1.38 ng/ml; P=0.111), either in participants with or without IR (91.84±1.28 vs. 91.84±1.45 ng/ml; P=0.971; Fig. 1). No statistically significant difference was demonstrated in plasma visfatin levels between the PCOS-IR (92.28±20.05 ng/ml), PCOS non-IR (108±44.28 ng/ml), control IR (96.83±34.06 ng/ml) and control non-IR (91.58±33.22) subgroups (P=0.467, among four subgroups; Fig. 2).

The plasma visfatin levels did not correlate with BMI, WHR, T, LH/FSH, FINS and blood lipid levels, although positive correlation with HOMA-IR was exhibited in the control IR patients (r=0.717; P=0.03) and negative correlation was exhibited with FPG in the PCOS non-IR patients (r=−0.641; P=0.046). However, the limited sample size did not allow a reliable multivariate analysis to be performed between the subgroups.

### Comparison of visfatin mRNA expression

Visfatin mRNA expression levels in the PBMCs of the PCOS patients was analogous to that of the non-PCOS participants (0.033±0.030 vs. 0.028±0.024; P=0.713). No significant differences were identified in the mRNA expression levels of visfatin in PBMCs between the IR and non-IR participants (0.0247±0.0248 vs. 0.036±0.028; P=0.394). Furthermore, no statistically significant difference in visfatin mRNA expression levels was demonstrated in PBMMs in the participants with and without PCOS (0.061±0.065 vs. 0.075±0.046; P=0.609), or with and without IR (0.053±0.043 vs. 0.083±0.064; P=0.064).

### Comparison of visfatin gene expression

Visfatin gene expression in PBMMs was greater than that observed in PBMCs of the non-PCOS participants (P=0.014), however, was not significantly increased in the PCOS patients (P=0.21), IR patients (P=0.06) or the non-IR participants (P=0.064).

## Discussion

As expected, the difference in hormone levels between the subgroups was comparable to the biochemical activity of PCOS. Although HOMA-IR and FINS were greater in the IR patients, the FPG levels were in the normal range, indicating that the function of the pancreas in these patients remained in the compensatory stage. Fallopian tube infertility patients were enrolled as control subjects in the present study, thus, the PCOS patients in the experimental group were younger. The DBP of the PCOS patients was greater than that of the control subjects, which may be the result of a disturbance in lipid metabolism and endothelial dysfunction that frequently occurs in PCOS. The BMI of the patients in the subgroups was normal, thus, the BMI was not adjusted in the PCOS and control groups. Triglyceride levels were markedly higher in the IR patients due to the increased production of triglycerides in the liver, combined with the reduced activity of lipoprotein lipase.

No difference in the levels of plasma visfatin was observed in the normal weight participants with and without PCOS or in the participants with and without IR, which was consistent with the results of previous studies (19,20). Increased levels of plasma visfatin have been demonstrated in other previous studies, however, this may have been induced by the confounding interference of obesity (10–14,17,18). In the present study, plasma visfatin levels negatively correlated with FPG, however, positively correlated with HOMA-IR in the PCOS non-IR and control-IR participants, respectively. Consistent with this, a positive correlation between plasma visfatin levels and HOMA-IR has been observed in previous studies (11,17,21,22). Visfatin was reported to bind to the insulin receptor via a tyrosine kinase and phosphorylate/activate the signaling pathway, performing insulin-like activities (9). In the present study it was hypothesized that PCOS and IR may play contrary roles, thus, no correlation was observed between visfatin and HOMA-IR in the PCOS-IR patients; however, the detailed mechanism was unclear.

When compared with a control group, increased visfatin gene expression was identified in omental adipose tissue and in mononuclear cells of PCOS patients (17,25). However, no significant difference in visfatin gene expression in PBMCs and PBMMs were observed between the PCOS and non-PCOS or IR and non-IR participants in the present study. These negative results may be due to a number of reasons. Firstly, tubal infertility patients were enrolled as controls in the present study. The majority of tubal infertility cases may have been induced by chronic pelvic inflammation, and visfatin levels may increase in patients with inflammation. Therefore, the visfatin levels of tubal infertility patients may also increase in a comparable manner to that of PCOS patients. Secondly, varying gene levels of visfatin were observed between cells in the peripheral blood and tissue (30). Visfatin mRNA expression levels in the PBMMs and PBMCs did not differ in the present study. A previous study indicated that the mRNA expression levels of visfatin in PBMCs did not correlate with the expression that was observed in the omental adipose tissue. Therefore, it was hypothesized that the gene expression of macrophages, infiltrated in adipose tissue, may be different to that of PBMMs *in vitro*. The inconsistencies that exist between macrophages and PBMMs can be explained by *in situ* stromal elements, including inflammatory cytokines, contributing significantly to the production of visfatin. Therefore, future studies are required to identify the role of visfatin in the pathogenesis of IR and PCOS on a tissue level, including adipose and ovary tissues. Although the sample size in the present study was small, a power calculation based on the data from the present study may be performed for future studies.

In conclusion, the plasma level of visfatin was not observed to increase in the normal weight participants with PCOS or IR and no correlation was observed. Visfatin gene expression levels observed in the PBMCs and PBMMs were not elevated in the normal weight PCOS subjects or the normal weight IR patients. Thus, further investigation regarding the role of visfatin in the pathogenesis of PCOS or IR should examine macrophages in the tissues, rather than in the peripheral blood.

## Figures and Tables

**Table I:** Clinical, endocrine and metabolic characteristics of the participants.

Indexes	PCOS IR (n=11) mean (SD)	PCOS non-IR (n=10) mean (SD)	Control IR (n=9) mean (SD)	Control non-IR (n=12) mean (SD)	P-value
Age (years)	25.09 (4.78)	24.7 (3.86)	29.22 (6.92)	30.08 (5.25)	0.043
Menarche age (years)	13.73 (1.79)	13.5 (2.32)	12.67 (1.23)	13.58 (1.56)	0.185^a^
SBP (mmHg)	110 (6.33)	107.7 (12.72)	97.56 (7.04)	104.58 (3.34)	0.471^a^
DBP (mmHg)	77.55 (7.23)	68.9 (8.36)	63.89 (4.17)	68.33 (7.79)	0.021^a^
Height (m)	1.55 (0.04)	1.57 (0.06)	1.56 (0.05)	1.55 (0.073)	0.857
Weight (kg)	57.55 (10.51)	49.86 (4.51)	53.03 (8.65)	48 (5.87)	0.206^a^
BMI (kg/m^2^)	23.97 (4.43)	20.31 (1.05)	21.71 (3.66)	20.06 (2.6)	0.311^a^
Waist (cm)	77.91 (10.44)	65.6 (3.86)	72.44 (11.31)	69.58 (9.4)	0.636^a^
Hip (cm)	90.55 (6.96)	87 (3.62)	89.67 (4.66)	86.17 (3.35)	0.454^a^
WHR	0.86 (0.07)	0.76 (0.05)	0.8 (0.09)	0.81 (0.09)	0.654^a^
E_2_ (pg/ml)	69.22 (17.85)	80.13 (26.55)	67.68 (35.06)	78.38 (55.24)	0.735^a^
P (ng/ml)	1.03 (0.31)	1.16 (0.3)	1.7 (2.85)	0.78 (0.31)	0.137^a^
T (ng/ml)	0.89 (0.29)	0.77 (0.31)	0.46 (0.11)	0.51 (0.23)	0.001
LH (mIU/ml)	14.3 (5.11)	15.36 (8.86)	8.49 (5.72)	4.53 (1.83)	<0.001^a^
FSH (mIU/ml)	4.65 (1.12)	5.45 (1.22)	5.57 (1.59)	6.33 (1.34)	0.037
LH/FSH	3.1 (0.99)	2.78 (1.33)	1.91 (2.03)	0.75 (0.44)	<0.001^a^
PRL (ng/ml)	11.76 (4.31)	12.54 (3.93)	16.71 (9.1)	15.69 (5.64)	0.191
T_3_ (nmol/l)	2.34 (0.47)	2.06 (0.16)	2.08 (0.37)	2.02 (0.44)	0.236
T_4_ (nmol/l)	102.88 (22.02)	107.09 (19.52)	112.36 (21.02)	106.91 (18.46)	0.781
PTC (nmol/l)	559.62 (102.74)	591.55 (123.11)	577.74 (133.93)	521.59 (93.85)	0.501
DHEAS (μg/dl)	8.51 (2.87)	6.54 (2.75)	5.82 (1.83)	5.45 (2.05)	0.025
FPG (mmol/l)	5.45 (0.46)	4.97 (0.47)	5.22 (0.43)	5.15 (0.31)	0.085
FINS (mIU/l)	15.12 (4.66)	6.13 (1.15)	12.3 (2.69)	6.41 (1.6)	0.003^a^
HOMA-IR	3.66 (1.2)	1.35 (0.23)	2.84 (0.62)	1.48 (0.41)	<0.001
Tch (mmol/l)	4.14 (0.76)	4.19 (0.54)	4.4 (0.67)	4.72 (0.91)	0.246
TG (mmol/l)	1.02 (0.32)	0.65 (0.21)	1.12 (0.18)	0.83 (0.27)	0.001
LDL (mmol/l)	2.34 (0.71)	2.23 (0.51)	2.22 (0.86)	2.69 (0.82)	0.405
HDL (mmol/l)	1.45 (0.32)	1.57 (0.21)	1.54 (0.34)	1.77 (0.3)	0.085

a Indicates a statistically significant outcome following the Napierian logarithm transformation among four subgroups.

PCOS, polycystic ovary syndrome; IR, insulin resistance; SBP, systolic blood pressure; DBP, diastolic blood pressure; BMI, body mass index; WHR, waist to hip ratio; E_2_, estradiol; P, progesterone; T, testosterone; LH, luteinizing hormone; FSH, follicle-stimulating hormone; PRL, prolactin; T_3_, triiodothyronine; T_4_, thyroxine; PTC, plasma total cortisol; DHEAS, dehydroepiandrosterone sulfate; FPG, fasting plasma glucose; FINS, fasting insulin; HOMA-IR, homeostasis model assessment of insulin resistance; Tch, total cholesterol; TG, triglyceride; LDL, low density lipoprotein cholesterol; HDL, high density lipoprotein cholesterol.
